# From pilot to policy: Adoption of the National mHealth application EZKarta in Czechia

**DOI:** 10.1177/20552076261430059

**Published:** 2026-03-04

**Authors:** Petra Hospodková Petrová, Jan Bruthans, Michaela Ondrejková

**Affiliations:** 1 Department of Biomedical Technology, Faculty of Biomedical Engineering, 198859 Czech Technical University, Prague, The Czech Republic; 2 Faculty of Health Sciences, 48261 Technical University of Liberec, Liberec, The Czech Republic

**Keywords:** mHealth, digital health, usability, governance, interoperability, health policy

## Abstract

**Background:**

Mobile health (mHealth) applications are increasingly seen as essential components of healthcare digitalization, yet many national initiatives struggle to progress beyond pilot phases. In Czechia, the EZKarta mobile application was launched by the Ministry of Health as a secure digital gateway to vaccination records and preventive check-ups, marking a first step toward a national eHealth platform.

**Objective:**

This study provides an exploratory, analytically grounded insight into early user perceptions and stakeholder views on EZKarta during its pilot phase, focusing on institutional, governance, and user-level factors influencing sustainability and integration.

**Methods:**

A mixed-methods design was applied. Quantitative data from a national online survey (*n* = 209) were analyzed using nonparametric tests. The qualitative component included semistructured interviews with key stakeholders. Findings were integrated through joint interpretation and thematic triangulation.

**Results:**

The mean usability score (UMUX = 33.2 ± 6.5) was significantly below the international benchmark (*p* < 2.2 × 10^−16^). Only 38% of users reported satisfaction, while 72% indicated willingness to use the application if integrated with their provider's clinical system. Triangulation of survey and interview data suggests that low engagement was driven primarily by limited functionality, lack of clinical system integration, and unclear perceived added value. Stakeholders highlighted fragmented governance as key barriers, while recognizing EZKarta's potential role in national digital health coordination.

**Conclusions:**

EZKarta exemplifies both the opportunities and constraints of mHealth adoption in transitional health systems. Stronger institutional coordination and transparent communication are essential for long-term relevance. The findings may inform policymakers in Central and Eastern Europe.

## Introduction

Mobile health (mHealth) technologies have become a global pillar of digital health strategies, with growing expectations to improve access, continuity of care, and patient engagement across diverse health systems worldwide. However, despite rapid technological development and widespread smartphone penetration, sustained mHealth adoption remains uneven across countries and regions. Global evidence consistently points to structural and sociotechnical barriers, including limited usability, insufficient integration into clinical workflows, privacy and trust concerns, fragmented governance, and difficulties in scaling pilot initiatives into routine care. These findings indicate that mHealth implementation challenges are not primarily technological, but systemic in nature.^1,2^

Within this global context, digital health has become an integral part of modern healthcare in Europe. mHealth applications support cardiac rhythm monitoring, medication adherence, and early disease detection, creating new opportunities for prevention and continuity of care. However, their implementation across Europe remains uneven.^3^ Although many countries have defined ambitious strategies, practice often lags behind, revealing a persistent gap between digital policy and healthcare delivery.^4–6^

During the past decade, the European Union has invested heavily in e-prescriptions,^7^ immunization registries, patient portals, mobile applications,^8^ and cross-border information exchange.^9^ Progress, however, has been slowed by outdated IT systems, fragmented governance, and poor interoperability.^10^ Considerable disparities remain: Estonia, Belgium, Denmark, and Finland are often cited as success stories, whereas Poland and Hungary continue to face governance and structural barriers.^11^

The Czech Act on Electronic Healthcare (No. 325/2021 Coll.) accelerated digitalization through e-prescriptions, e-sick leave, and teleconsultations.^12^ Yet, mHealth adoption remains weak due to institutional immaturity, inconsistent strategic direction, and fragmented policy environments.^8^

The EZKarta mobile application, developed by the Czech Ministry of Health, represents a key step in the digitalization of healthcare services in Czechia. Launched in 2020 as a secure digital gateway, it initially allowed citizens to access their vaccination records (including COVID-19 data) and those of their dependants, available for both Android and iOS platforms. Originally introduced as Tečka (“Full Stop”), it was later rebranded as EZKarta (Electronic Health Card) to reflect plans for expansion into a comprehensive eHealth platform. In August 2025, EZKarta was expanded with a “My Health” section featuring overviews of completed screening examinations and preventive check-ups; however, the present study evaluated the pre-expansion pilot version, and subsequent updates are described for contextual purposes only and were not evaluated. Planned developments include integration with central health registers, electronic communication with providers, and a searchable map of healthcare facilities, followed by appointment scheduling, preventive reminders, and secure sharing of medical documentation and laboratory results. Although still limited in scope, EZKarta represents an important step toward aligning national mHealth policy with real-world implementation and patient engagement.

European reviews confirm that most mHealth initiatives remain small, donor-funded pilots with limited scalability.^13,14^ Despite growing attention to digital health, empirical evidence on mHealth adoption in postsocialist healthcare systems remains limited, particularly in Czechia.

This study was conceptually informed by the Normalization Process Theory (NPT),^15^ which explains how complex innovations become embedded into routine practice through four generative mechanisms: coherence (sense-making), cognitive participation (engagement), collective action (implementation), and reflexive monitoring (evaluation). Applying NPT provided a structured lens for interpreting stakeholder perspectives and user adoption factors, highlighting how institutional alignment, professional endorsement, and feedback loops shape the normalization of digital tools such as EZKarta within the healthcare system. The framework guided both the design of the interview guide and the integration of qualitative and quantitative findings.

Therefore, this study examines the adoption and perceived usability of EZKarta as a case study of mHealth deployment in a postsocialist context. Specifically, it aims to:

- explore stakeholder views on integration and ecosystem readiness using semistructured interviews and
- identify user perceptions, barriers, and motivators influencing mHealth adoption through a national survey.

By combining quantitative and qualitative insights, the study seeks to enhance understanding of sociotechnical and institutional factors shaping mHealth adoption in Central Europe. The findings are intended to inform national and European strategies aimed at moving from pilot projects to sustainable, interoperable, and inclusive digital health ecosystems.

## Methods

The study employed a convergent mixed-methods design, integrating qualitative, and quantitative data to analyze institutional and user-level factors influencing EZKarta adoption in Czechia. The qualitative component explored the perspectives of stakeholders involved in digital health governance, while the survey quantified user experiences and perceived usability. Data were collected and analyzed in parallel, followed by joint interpretation to ensure complementarity and triangulation.^16^ The research was conducted in the first half of 2025, prior to the expansion of EZKarta with the “MyHealth” section.

Ethical approval was obtained from the Ethics Committee of the Faculty of Biomedical Engineering, Czech Technical University (No. C47/2025, 10 February 2025) in Supplemental material 1. Written informed consent was obtained from all participants from the qualitative component of the study Supplemental material 2.

### Qualitative component

The qualitative interviews were designed to address aim (i) by exploring stakeholder views on EZKarta's integration within the national digital health ecosystem, perceived institutional readiness, and sustainability considerations during the pilot phase.

#### Research team and reflexivity

Interviews were conducted by a multidisciplinary team with expertise in biomedical engineering, health policy, and digital health. The lead interviewer (female, PhD in health systems management) and the co-interviewer (male, PhD in biomedical informatics) had prior experience in eHealth research. Both reflected on potential biases and documented their assumptions before and during data collection to preserve neutrality. They had no professional relationship with participants and no involvement in the EZKarta project.

#### Study design and participants

An interpretivist approach was used, recognizing that stakeholder perceptions are context-dependent. Semistructured interviews explored the implementation, usability, and strategic relevance of EZKarta, following COREQ standards.^17^ Purposive sampling^18^ was employed to ensure a balanced representation of perspectives. Twelve experts participated across four stakeholder groups (Table 1).

**Table 1.** Overview of stakeholder groups and participant distribution.

Stakeholder group	Description	Number (*n*)
Policy Makers (PM)	Ministry of Health, eHealth and interoperability departments	3
Healthcare Providers (HP)	General practitioners and pediatricians involved in pilot use	3
Technical Experts (IT)	Specialists in data security, interoperability, and digital infrastructure	3
Academia/Researchers (AR)	Scholars in digital health policy and evaluation	3

Eligibility criteria included ≥5 years of experience in healthcare digitalization and involvement in relevant projects. In addition, all participants had direct professional exposure to EZKarta, either through pilot use, technical, or interoperability-related work, policy coordination at the national level, or research and evaluation activities focused on EZKarta or closely related national eHealth initiatives. Participants were recruited via professional networks, ministry working groups, and public events. Participants occupied senior or expert-level positions within their respective stakeholder groups, represented both public and private sector institutions, and had a median of approximately 10 years (range 5–25 years) of professional experience in healthcare digitalization. No invited participants declined participation.

Data saturation was reached after 12 interviews, with no participants withdrawing. Interviews were conducted online via Microsoft Teams between March and April 2025, lasting 45 to 60 min.

The interview guide, developed based on the literature,^6,14,19,20^ covered five domains: (1) the purpose of EZKarta within the national strategy; (2) institutional and regulatory enablers or barriers; (3) user engagement and data trust; (4) technical integration; and (5) sustainability. Participants were informed in advance about the general focus and thematic domains of the interview; however, the full interview guide was not shared prior to the interview in order to preserve spontaneity and minimize response conditioning. The guide was piloted with two experts. The complete interview guide is available in Supplemental material 3. All interviews were audio-recorded with consent and transcribed verbatim. Data were anonymized and stored securely in accordance with General Data Protection Regulation (GDPR).

#### Data analysis

Reflexive thematic analysis^21^ was conducted in MAXQDA 24 in four stages: familiarization, inductive coding, theme development, and interpretation. Two researchers coded the data independently; discrepancies were resolved through discussion and verified by a third reviewer. Validity was enhanced through triangulation, peer debriefing, and member checking.

### Quantitative component

The questionnaire-based survey was designed to address aim (ii) by identifying user perceptions, barriers, and motivators influencing EZKarta adoption, with a specific focus on perceived usability and usefulness during the pilot phase.

#### Study design and participants

The cross-sectional survey (21 January–21 April 2025) assessed the usability and perceived value of EZKarta among users and nonusers. The questionnaire was distributed via the Ministry of Health, Patient Hub, the National Health Information Portal (NZIP), and partner initiatives such as Loono's Preventivka application, using publicly accessible channels without paid, targeted, or algorithmic promotion; consequently, the total number of individuals exposed to the survey invitation could not be determined. Inclusion criteria were: age ≥18 years, Czech residency, and awareness of EZKarta. Awareness was assessed using a screening item asking whether respondents had heard of the EZKarta application prior to participation. Users were defined as respondents who had downloaded and used the application, whereas nonusers were aware of EZKarta but had never downloaded or used it.

The questionnaire included four sections: demographics, usage, usability, and perceived usefulness. The median completion time recorded by the survey platform was 8 minutes. Prior to public dissemination, the questionnaire was reviewed by experts with professional experience in digital health and eHealth implementation and pilot-tested (*n* = 5) to ensure clarity, content validity, and internal consistency (Cronbach's *α* = 0.86). Usability was measured using the Usability Metric for User Experience (UMUX),^22^ a four-item, seven-point Likert scale standardized to a 0 to 100 scale for comparability with the System Usability Scale (SUS)^23^; however, UMUX is not identical to SUS, and the commonly cited SUS reference value of 68 was therefore used as a pragmatic point of comparison rather than a definitive pass/fail usability threshold**.** The full survey instrument is provided in Supplemental material 4.

#### Research hypotheses

Three core hypotheses, along with their rationale, were formulated (Table 2).

**Table 2.** Research hypotheses and theoretical rationale.

Code	Hypothesis	Rationale
H1	The mean UMUX score for EZKarta exceeds the reference usability threshold of 68 (SUS equivalent)	Tests overall user satisfaction and usability
H2	Younger users (18–35 years) evaluate the application more positively than older users (36 + years).	Reflects the digital literacy gap and age-related usability differences
H3	Users assign higher perceived usefulness to existing and proposed functionalities (e.g. digital vaccination records, e-prescriptions) compared to nonusers	Assesses acceptance and adoption potential

#### Data analysis

Data were processed in IBM SPSS v29 and R 4.3.2. Descriptive statistics were used to summarize demographics and usability. Normality was tested using the Shapiro–Wilk test (*p* < 0.05); nonparametric tests were applied:

- H1: one-sample Wilcoxon signed-rank test
- H2, H3: Mann–Whitney U tests

Although nonparametric tests were applied due to deviations from normality, mean ± SD are reported to facilitate comparability with prior digital health usability studies, while medians are provided where available.

Statistical significance was set at *α* = 0.05; effect sizes (*r*) were reported for selected primary comparisons, and 95% confidence intervals (CIs) were provided where applicable. Integration of quantitative and qualitative findings enabled triangulation and strengthened interpretive validity.

The integration of qualitative and quantitative findings followed a side-by-side comparison approach as recommended by Creswell et al.^16^ Themes and statistical findings were compared iteratively to identify convergent, complementary, and divergent evidence across data strands. Triangulation occurred in three stages: (1) initial comparison of descriptive statistics and preliminary codes, (2) cross-validation of emergent themes with quantitative indicators, and (3) joint interpretation during the final analytic synthesis. This process enhanced methodological rigor and ensured that interpretations reflected both user-level and institutional perspectives.

## Results

### Qualitative component

The qualitative component drew on 12 semistructured interviews representing policymaking, healthcare provision, technical, and academic perspectives. Inductive analysis in MAXQDA identified themes grouped into three domains: (1) institutional and governance context, (2) user and communication factors, and (3) strategic outlook and sustainability. Thematic frequencies are summarized in Tables 3 and 4. Findings are interpreted as exploratory, reflecting stakeholder insights during the EZKarta pilot rather than supporting population-level generalizations.

**Table 3.** Distribution of main qualitative codes (coded segments).

Main theme (code)	Coded segments (*n*)	Share of total coded data (%)	Illustrative meaning
EZKarta (application-specific issues)	184	41.3	Practical experience with the pilot version, usability, data sharing, integration with GP systems
Digitalization of healthcare in Czechia	139	31.2	Broader policy and infrastructure context, legislation, interoperability
International comparison of digital health systems	50	11.2	References to Nordic models, EU best practices, and benchmarking
Key priorities for health-system digitalization (3–5 years)	33	7.4	Expectations for future development of national eHealth strategy
International inspiration & collaboration	22	4.9	Cross-border cooperation and European initiatives (e.g. MyHealth@EU, EHDS)
Professional roles & responsibilities	17	3.8	Distribution of responsibilities among actors, training and capacity-building needs
Total	445	100	—

**Table 4.** Presence of subthemes by stakeholder group.

Domain	Subtheme	PM	HP	IT	AR
Institutional & governance context	Fragmented responsibility and unclear accountability	●	●	●	○
	Policy inertia and limited coordination	●	●	○	●
	Data ownership and privacy ambiguity	●	○	●	○
	Limited integration with providers’ clinical systems	○	●	●	○
User & communication factors	Low public awareness and promotion	●	●	○	●
	Limited current functionality (vaccination record only)	○	●	●	○
	Trust and data-protection concerns	●	●	●	○
	Digital-literacy barriers (older adults)	○	●	○	●
Strategic outlook & sustainability	Long-term funding and maintenance model	●	○	○	●
	Alignment with the European Health Data Space (EHDS)	●	○	●	●
	Provider motivation and participation	●	●	○	●
	Continuous evaluation and user feedback	○	●	●	●

● Presence of the subtheme in the group's interviews; ○ not prominent in that group's interviews).

AR: Academia/Researchers; HP: Healthcare Providers; IT: Technical Experts; PM: Policy Makers.

#### Institutional and governance context

Through interviews, participants described a fragmented policy environment marked by overlapping competences and slow coordination. Several policymakers noted that responsibility for digital health is distributed across ministries and agencies, without a stable steering structure. As one policymaker put it, “*We have plenty of pilot projects but no real coordination; every institution runs its own initiative*” (PM2). Another added that, despite political interest, “*the agenda still depends on individual enthusiasm rather than an institutional mandate*” (PM1).

Fast Healthcare Interoperability Resources (FHIR) is a widely used interoperability standard that participants referenced when discussing technical integration. “*Standards like FHIR are recognized, but no one is clearly responsible for ensuring their use,*” stated one expert (IT1). Others warned that the absence of accountability for data governance complicates decision making and contributes to stakeholder fatigue.

Providers took a pragmatic view: while welcoming innovation, they expressed doubts about the efficiency of a system not integrated with their existing software. As one general practitioner explained, “*Unless it's connected to the system we already use, it just means extra work for physicians*” (HP1).

#### User and communication factors

This domain captured issues of communication, usability, and trust. Nearly all respondents agreed that public awareness of EZKarta was low during the pilot phase—interpreted not as user resistance but as a result of insufficient communication and professional endorsement. As one academic observed, “*Many people don’t even know this app exists; even among doctors it's rarely discussed*” (AR1). A policymaker added, “*Without clear guidance from GPs, citizens simply don’t know why they should use it*” (PM3).

Most participants found the interface intuitive but considered its limited functionality a major drawback. A healthcare provider remarked, “*It's a good foundation, but without e-prescriptions and medical records, people won’t use it*” (HP2). Another noted, “*Users expect one place where they see everything—from vaccinations to test results*” (HP3).

Concerns about data protection were frequent, though perceived mainly as communication rather than technical issues. As one IT specialist pointed out, “*Technically it's safe, but users need to understand who accesses their data and why*” (IT3). An academic added, “*Security is rarely the problem; the problem is how it's explained*” (AR2).

Digital literacy challenges, particularly among older adults, were also noted. Participants agreed that educational campaigns and clear communication could reduce digital exclusion. Overall, effective communication and perceived usefulness—rather than technical design—were viewed as key drivers of adoption in early implementation.

#### Strategic outlook and sustainability

Stakeholders reflected on EZKarta's strategic potential and future direction. Most described it as a pilot enabling “learning by doing” rather than a mature platform. As one policymaker explained, “*EZKarta could become the entry point to personal health data, but there is no clear vision for linking it with European initiatives*” (PM1). Several noted that its symbolic importance currently exceeds its technical maturity.

Discussions on sustainability centered on funding, evaluation, and provider engagement. While no financial shortages were reported, participants pointed to the absence of a mechanism for long-term maintenance or iterative development. An IT expert noted, “*Projects often start with enthusiasm and end when the pilot funding stops*” (IT2).

Academics and practitioners alike stressed the need for continuous evaluation and feedback: “*Without continuous evaluation and user feedback, the application will stagnate*” (AR2). The most widely shared view concerned the role of healthcare providers as key adopters. As one clinician summarized, “*The key to success won’t be the technology itself, but whether providers actually use it*” (HP3).

Overall, EZKarta was seen not as a technological endpoint, but as a testing ground revealing the governance, communication, and resource conditions essential for sustained digital health transformation in Czechia.

### Quantitative component

A total of 357 respondents attempted to complete the questionnaire; 209 participants (120 women and 89 men) completed it in full, yielding a completion rate of 58.5%, as the total number of individuals exposed to the survey invitation could not be determined. Among them, 125 were active users of EZKarta and 84 were nonusers. Participants represented all regions of the Czechia, with a balanced age distribution (41% aged 18–35 years and 59% aged 36 years or older). Most respondents reported tertiary education and moderate to high digital literacy, reflecting the characteristics of the population likely to engage with national eHealth tools. Approximately one-fifth were healthcare professionals, providing insight into both professional and lay user perspectives.

A summary of the quantitative hypothesis testing is presented in Table 5.

**Table 5.** Summary of quantitative hypothesis testing.

Hypothesis	Tested variable/groups	Statistical test	*n*	Main findings	*p*-value	Result
H1	UMUX vs SUS benchmark (68)	Wilcoxon signed-rank (1-sample)	125	Mean = 33.20 (±6.53); Mdn = 34.83 < 68 reference value	<2.2 × 10^−16^*	Not supported
H2	Age groups (18–35 vs 36+) × UMUX	Mann–Whitney U	28/97	Mdn = 33.83 vs 35.00; no significant difference	0.87	Not supported
H3	Users vs Nonusers × Perceived usefulness	Mann–Whitney U	125/84	Significant for digital vaccination record, e-prescriptions, lab results, secure sharing, and medical history summary (users > nonusers)	<0.05*	Supported

All tests nonparametric (*α* = 0.05). Significant differences are indicated by an asterisk (*). Functional categories refer to items from the questionnaire on current and proposed EZKarta features.

#### Overall usability (H1)

Testing whether EZKarta's UMUX score exceeded the SUS benchmark (68) yielded a mean of 33.20 ± 6.53 (median 34.83). The Wilcoxon signed-rank test indicated a significantly lower score (*p* < 2.2 × 10**^−^**^16^), indicating that usability perceptions were substantially below this pragmatic reference point. The corresponding effect size (*r* = 0.74) indicates a large deviation from the benchmark according to Cohen's criteria,^24^ demonstrating that the usability deficit is not only statistically significant but also substantial in practical terms. This suggests that major design and communication improvements are required before the application's broader deployment. Open-ended comments aligned with this result, citing limited functionality as the main reason.

#### Age-related differences (H2)

The Mann–Whitney U test found no significant age effect (*U* = 2925, *p* = 0.87); median scores were similar (18–35 years = 33.83; 36+ years = 35.00). Age, therefore, did not influence perceived usability.

#### Perceived usefulness (H3)

Users rated usefulness significantly higher than nonusers (*p* < 0.05) for five features: digital vaccination record, e-prescriptions, lab results, secure data sharing, and medical history summary—indicating that active experience increases perceived utility, particularly for interoperable functions.

Table 5 summarizes the outcomes of the three hypotheses, showing that usability (H1) fell significantly below the applied reference benchmark, while perceived usefulness (H3) differed significantly between users and nonusers.

## Discussion

Despite many efforts to unify processes across EU countries, healthcare remains largely under the sole governance of individual member states. Recent initiatives to digitalize healthcare are also mostly limited to unplanned, emergency cross-border care,^25^ aiming only to interlink independently developed systems without attempting to influence their fundamental design.

Embedding mHealth in such a decentralized and fragmented system is challenging and often results in isolated pilot projects and limited diffusion of innovation.^3^ In this context, the lack of integration of EZKarta with providers’ clinical systems should be understood as a consequence of its pilot-stage implementation combined with fragmented governance structures, heterogeneous provider IT infrastructures, and the absence of a centralized mandate to enforce interoperability. Public trust is equally decisive. Beyond technical capacity, citizens’ confidence in data protection, transparency, and usefulness determines engagement. Privacy concerns, low digital literacy, and technological anxiety among older adults remain major barriers.^26–28^ Without clear reimbursement and regulatory mechanisms, many solutions fail to progress beyond the pilot stage.^29–33^ NPT explains how innovations become embedded in practice through coherence, cognitive participation, collective action, and reflexive monitoring.^15^ Implementation science extends this approach by linking barriers—such as role ambiguity, workflow disruption, and regulatory uncertainty—to adoption outcomes.^19,34,35^ Additional obstacles include digital exclusion, insufficient stakeholder engagement, and lack of user training,^20^ whereas sustainable implementation depends on leadership, participatory co-design, secure financing, and consistent evaluation.^36^

Beyond governance fragmentation, the findings underscore how mHealth adoption depends not only on political commitment but also on the maturity of interinstitutional collaboration and stakeholder alignment. In postsocialist health systems such as Czechia, where hierarchical decision-making traditions coexist with newly decentralized structures, digital health policies often evolve faster than the administrative mechanisms required to implement them. This institutional lag produces a “pilot paradox,” referring to situations in which innovation is encouraged through pilot projects but rarely sustained due to missing structural conditions. Thus, the findings suggest that this experience reflects a broader regional need to move from project-based digitalization toward coherent, system-level governance, a concern repeatedly emphasized by interviewed stakeholders when reflecting on sustainability beyond the pilot phase.

User and communication factors further illuminate the complexity of mHealth integration. Both interview and survey data highlighted limited public awareness and modest perceived usefulness of EZKarta during its pilot phase. The below-benchmark UMUX score of ≈33 suggests that usability alone cannot compensate for insufficient communication or narrow functionality, indicating that technical design represents only one dimension of adoption, alongside communication and perceived value as highlighted in both survey and interview data. This aligns with previous studies indicating that perceived value and trust, rather than interface quality, are the key drivers of sustained engagement.^19,27^

UMUX scores standardized to a 0 to 100 scale are commonly interpreted in relation to SUS reference values for pragmatic comparison.^23^ Studies of mature national patient portals, particularly in Nordic and Western European countries, typically report SUS-equivalent usability scores in the range of approximately 65 to 80, reflecting broad functional scope, clinical integration, and sustained provider endorsement.^37^ Hospital-based or regional mHealth applications often achieve intermediate scores (approximately 55–70).^38^ Scores below 50 are commonly interpreted as indicating poor or unacceptable usability in SUS-based evaluations.^39^ The EZKarta usability score is substantially lower than those reported for mature national platforms.

Importantly, comparative studies of national mHealth implementations suggest that low or moderate usability scores alone do not fully explain adoption outcomes. For instance, Hägglund et al.^37^ observed that national patient portals in the Nordic countries achieved higher adoption only when provider endorsement and clear communication accompanied technical rollout, rather than usability alone. Ramdani et al.^38^ found in hospital-based mHealth adoption that institutional readiness, stakeholder engagement, and data governance overshadowed interface design as determinant factors. These parallels suggest that EZKarta's challenges are not unique but reflect broader systemic issues common to transitional digital-health ecosystems.

Participants in this study consistently linked trust and clarity about data handling to user acceptance. Although technical experts confirmed compliance with data-protection standards, users’ uncertainty about who can access their information points to a transparency gap rather than a security deficit. Addressing this gap was described by participants as requiring multichannel communication, co-branding with trusted healthcare institutions, and visible involvement of healthcare providers as mediators of trust and legitimacy.

Digital literacy emerged as another key determinant. This low literacy is not limited to patients; providers also require structured training to interpret and communicate digital data effectively. Without this, stakeholders cautioned that mHealth risks reinforcing rather than reducing inequalities in access and understanding.

The strategic outlook and sustainability domain revealed both optimism and caution. Stakeholders perceived EZKarta as a symbolic achievement—a visible manifestation of national digital health progress—yet also as a pilot without guaranteed continuity. Absence of long-term funding mechanisms or iterative evaluation cycles was a recurring concern. Stakeholders perceived integration of EZKarta with cross-border infrastructures like MyHealth@EU as a potential pathway to transform it from a national application into an interoperable gateway for European data exchange. Participants suggested that such integration could enhance both technical value and citizens’ trust in its long-term relevance, although this remains contingent on governance and communication arrangements.

Taken together, the mixed-methods findings portray EZKarta as an evolving platform situated between technological potential and systemic inertia. Quantitative data reveal low initial usability, yet qualitative insights highlight a latent willingness among users and professionals to engage—provided the system becomes functionally rich, trustworthy, and well-governed. For policymakers, the key lesson is that digital transformation requires more than technical solutions; it demands stable leadership, intersectoral coordination, and sustained communication. Interpreting these findings through the lens of NPT clarifies why EZKarta has not yet become embedded in routine practice. Coherence remained partial: while stakeholders recognized EZKarta's strategic role in national digitalization, users reported limited understanding of its purpose beyond vaccination records, reflected in low perceived usefulness and usability. Cognitive participation primarily concerned healthcare professionals rather than patients; weak provider endorsement and unclear professional incentives constrained broader engagement. Collective action was impeded by limited integration with clinical systems, unclear governance responsibilities, and perceived workflow disruption. Finally, reflexive monitoring was underdeveloped, as stakeholders highlighted the absence of systematic evaluation, user feedback loops, and long-term sustainability mechanisms. Together, these gaps across all four NPT mechanisms help explain why EZKarta has remained at the pilot stage despite technical readiness.

This study has several limitations. Both components of the mixed-methods design relied on voluntary participation, introducing potential self-selection bias toward respondents with higher digital literacy and interest in eHealth. The sample size of the qualitative component (*n* = 12) was appropriate for exploratory analysis but limits transferability to broader stakeholder populations. In addition, the cross-sectional design captures perceptions at a single point in time and does not allow assessment of changes in usability or engagement as the application evolves, introducing potential cross-sectional bias.

Usability was assessed using a self-reported instrument (UMUX), which reflects perceived rather than observed user performance and may be influenced by expectations, prior experience, or limited exposure to the application's functionality. As such, the usability results should be interpreted as indicative of early user perceptions rather than objective measures of task efficiency or effectiveness.

The study did not include healthcare policymakers beyond the national level, excluding perspectives from regional authorities and patient associations, which could further illuminate contextual factors influencing uptake. The cross-sectional survey captured perceptions at a single point in time only—before the August 2025 “My Health” expansion—and therefore cannot assess longitudinal changes in usability or adoption.

Future studies should overcome these limitations by expanding stakeholder diversity and applying longitudinal or experimental designs to evaluate behavioral shifts following major updates. Further research should examine interoperability performance, cost-effectiveness, and clinical impact as EZKarta integrates with national registries and the European Health Data Space. Continuous monitoring of user experience, provider engagement, and public trust will be essential to ensure that the platform evolves from a pilot initiative into a sustainable component of Czech digital health ecosystem.

### Reflexivity and generalizability

The research team was academically affiliated and independent of the Ministry of Health and other institutions involved in the EZKarta project. This external analytical position reduced potential bias but may have limited contextual familiarity with internal policy dynamics. The qualitative findings therefore reflect an outsider analytical perspective, emphasizing perceived patterns rather than insider processes. While the study was conducted within Czechia, its findings are transferable to other transitional healthcare systems facing similar institutional and governance challenges in national mHealth implementation.

### Policy and practice implications

The findings suggest that the long-term success of EZKarta and similar national mHealth initiatives depends less on technical optimization and more on institutional stewardship and communication coherence. Stakeholders emphasized the importance of embedding mHealth tools into provider workflows and national prevention programs to enhance legitimacy and sustained use. Based on these findings, a plausible policy approach discussed by participants would involve prioritizing interoperability with electronic health records, strengthening strategic communication to build trust, and establishing continuous evaluation mechanisms, potentially coordinated by an independent or semi-independent body. Lessons from this case can inform digital health policy in other transitional healthcare systems seeking to move from pilot projects to scalable, citizen-oriented platforms.

## Conclusion

The EZKarta initiative illustrates both the opportunities and persistent challenges of implementing national mHealth solutions within a fragmented health system. While its early adoption phase revealed low usability and limited functionality, the study also highlighted broad stakeholder support and a clear readiness for digital transformation. Bridging the gap between pilot and policy will require coordinated governance, stable financing, and sustained engagement of healthcare providers and citizens alike. Ensuring EZKarta long-term success will depend on continuous evaluation, transparent communication, and a commitment to inclusivity in digital health innovation.

## Supplemental Material

sj-pdf-1-dhj-10.1177_20552076261430059 - Supplemental material for From pilot to policy: Adoption of the National mHealth application EZKarta in Czechia Supplemental material, sj-pdf-1-dhj-10.1177_20552076261430059 for From pilot to policy: Adoption of the National mHealth application EZKarta in Czechia by Petra Hospodková Petrová, Jan Bruthans and Michaela Ondrejková in DIGITAL HEALTH

sj-pdf-2-dhj-10.1177_20552076261430059 - Supplemental material for From pilot to policy: Adoption of the National mHealth application EZKarta in Czechia Supplemental material, sj-pdf-2-dhj-10.1177_20552076261430059 for From pilot to policy: Adoption of the National mHealth application EZKarta in Czechia by Petra Hospodková Petrová, Jan Bruthans and Michaela Ondrejková in DIGITAL HEALTH

sj-pdf-3-dhj-10.1177_20552076261430059 - Supplemental material for From pilot to policy: Adoption of the National mHealth application EZKarta in Czechia Supplemental material, sj-pdf-3-dhj-10.1177_20552076261430059 for From pilot to policy: Adoption of the National mHealth application EZKarta in Czechia by Petra Hospodková Petrová, Jan Bruthans and Michaela Ondrejková in DIGITAL HEALTH
