# Supplementary material for: From pilot to policy: Adoption of the National mHealth application EZKarta in Czechia
Source: Digit Health. 2026 Mar 4;12:20552076261430059. doi: 10.1177/20552076261430059 (PMC12961103; doi:10.1177/20552076261430059)
Supplement: sj-pdf-1-dhj-10.1177_20552076261430059 - Supplemental material for From pilot to policy: Adoption of the National mHealth application EZKarta in Czechia [file sj-pdf-1-dhj-10.1177_20552076261430059.pdf]

Supplementary Material 1

ČESKÉ VYSOKÉ UČENÍ TECHNICKÉ V PRAZE
Fakulta biomedicínského inženýrství
nám. Sítná 3105, 272 01 Kladno

Žádost o projednání výzkumného projektu v etické komisi FBMI ČVUT

Application for approval of a research project by FBMI CTU Institutional Ethical/Review Board

Název projektu: Mobilní aplikace EZKarta: Zpětná vazba uživatelů a potenciál pro další rozvoj
Name of the project: Mobile Application EZKarta: User Feedback and Potential for Further Development

Hlavní řešitel projektu (Jméno, pracoviště, e-mail): Bc. Michaela Ondrejková, ondremic@fbmi.cvut.cz
Vedoucí kvalifikační práce (Jméno, pracoviště, e-mail): Ing. Petra Hospodková, Ph.D., MBA Katedra biomedicínské techniky, FBMI ČVUT v Praze, hospopet@fbmi.cvut.cz

Stručný popis projektu (do 100 slov):
Účelem této studie v rámci diplomové práce je analyzovat názory a postoje k implementaci aplikace EZKarta z perspektivy klíčových zainteresovaných stran, jako jsou zástupci Ministerstva zdravotnictví, odborníci na IT a vývojáři. Výzkum se zaměřuje na identifikaci přínosů, výzev a možných rizik spojených s implementací této aplikace, která má sloužit jako nástroj pro správu a sdílení zdravotnických dat o pacientovi. Studie bude realizována formou polostrukturovaných rozhovorů vedených online prostřednictvím platformy MS TEAMS. Cílem výzkumu je mimo jiné zhodnotit připravenost jednotlivých aktérů na zavedení EZKarty, analyzovat technologické aspekty a identifikovat oblasti, které vyžadují další rozvoj.

Charakter projektu:
Grantová úloha (název agentury):
☑ Výzkum výzkumného týmu (specifikace):
Kvalifikační práce (specifikace): DP
Jiné:

Seznam přikládaných dokumentů:
- sylabus projektu
- informovaný souhlas vč. informace pro subjekt hodnocení
- strukturovaný životopis hlavního řešitele, je-li na FBMI externistou
- jiné (prosíme vypsat):

V Kladně dne 20.1.2025

[signature]
podpis hlavního řešitele

Vyjádření souhlasu etické komise FBMI ČVUT

FBMI CTU Institutional Ethical/Review Board approval

Projekt byl schválen etickou komisí FBMI ČVUT dne: 27.2.2025 **platný do:** 9/2025
pod číslem: C 68/2025

Etická komise FBMI ČVUT v Praze, ve složení Mgr. Martina Dingová Šliková, Ph.D. (předsedkyně), prof. Ing. Karel Roubík, Ph.D., RNDr. Táňa Jarošíková, CSc., doc. Ing. Petr Kudrna, Ph.D., MUDr. Tomáš Heřman a Ing. Lucie Šedzmáková, zhodnotila předložený projekt a neshledala žádné rozpory s platnými zásadami, předpisy a mezinárodními směrnicemi pro provádění biomedicínského výzkumu zahrnujícího lidské účastníky nebo laboratorní zvířata.

Řešitel projektu splnil podmínky nutné k získání souhlasu etické komise.

V Kladně dne 12.5.2025

ETICKÁ KOMISE
České vysoké učení technické v Praze
Fakulta biomedicínského inženýrství
nám. Sítná 3105
272 01 Kladno
razítko etické komise FBMI ČVUT

Mgr. Martina Dingová Šliková, Ph.D.
podpis předsedy etické komise

ČVUT v Praze
Fakulta biomedicínského inženýrství
nám. Sítná 3105
272 01 Kladno

tel.: (+420) 224 358 419
fax: (+420) 312 608 204
www.fbmi.cvut.cz

IČ: 68407700
DIČ: CZ68407700
Bankovní spojení: KB Praha 6
č.ú. 27-7380010287/0100
