# Supplementary material for: From pilot to policy: Adoption of the National mHealth application EZKarta in Czechia
Source: Digit Health. 2026 Mar 4;12:20552076261430059. doi: 10.1177/20552076261430059 (PMC12961103; doi:10.1177/20552076261430059)
Supplement: sj-pdf-2-dhj-10.1177_20552076261430059 - Supplemental material for From pilot to policy: Adoption of the National mHealth application EZKarta in Czechia [file sj-pdf-2-dhj-10.1177_20552076261430059.pdf]

Supplementary Material 1

Czech Technical University in Prague

Faculty of Biomedical Engineering

nám. Sítná 3105, 272 01 Kladno

Application for Approval of a Research Project by the FBMI CTU Institutional Ethical Review Board

Project Title: Mobile Application EZKarta: User Feedback and Potential for Further Development

Brief Project Description (up to 100 words): The objective of this study is to analyze opinions and attitudes regarding the implementation of the EZKarta application from the perspective of key stakeholders within the healthcare sector, including representatives of the Ministry of Health, healthcare providers, and IT developers. The aim of the research is to identify strengths, weaknesses, and risks associated with the implementation of the application, particularly in relation to data sharing and communication between patients and healthcare providers. Qualitative research methods are applied, specifically semi-structured interviews conducted via the MS Teams platform. The research focuses on assessing readiness for the deployment of EZKarta, analyzing technological aspects, and identifying obstacles or drivers for further development.

Ethical Approval

The research project was reviewed and approved by the Institutional Ethical Review Board of the Faculty of Biomedical Engineering, Czech Technical University in Prague. The review confirmed compliance with international ethical standards for biomedical research involving human participants.
