# Supplementary material for: From pilot to policy: Adoption of the National mHealth application EZKarta in Czechia
Source: Digit Health. 2026 Mar 4;12:20552076261430059. doi: 10.1177/20552076261430059 (PMC12961103; doi:10.1177/20552076261430059)
Supplement: sj-pdf-3-dhj-10.1177_20552076261430059 - Supplemental material for From pilot to policy: Adoption of the National mHealth application EZKarta in Czechia [file sj-pdf-3-dhj-10.1177_20552076261430059.pdf]

Supplementary Material 2

Informed consent will be granted implicitly within the recording of semi-structured interviews. Participants will be contacted by email (see text below). Alternatively, they may be contacted by telephone.

Dear Madam, Dear Sir,

We would like to invite you to participate voluntarily in a research study entitled:
“Mobile Application EZKarta: User Feedback and Potential for Further Development.”

The aim of this study is to obtain opinions, perspectives, and experiences of key experts involved in healthcare digitalization, with a particular focus on the technological and organizational aspects of the development of the EZKarta application.

Your participation would require approximately **50–60 minutes** of your time. Below, we provide you with basic information about the research.

Information

The study is based on semi-structured interviews conducted according to a predefined interview guide. During the interview, you will be asked questions focusing on your experience, your views on technical challenges and opportunities in the development of the EZKarta application, as well as on organizational and legislative aspects of the implementation of this platform within the healthcare system.

The interviews will be conducted via MS Teams and, with your consent, will be audio-recorded. Access to the online meeting will be provided via a link sent to the email address of your choice. The recording will be deleted after transcription into an anonymized text form, no later than 90 days after the interview, using the secure deletion tool *Eraser*.

Approximately 10 additional experts from the fields of IT and healthcare, who are directly involved in healthcare digitalization and the EZKarta application, will participate in the research.

Your participation is entirely voluntary, and no risks or harm are anticipated as a result of taking part in the study. At the same time, the study is not expected to bring any direct clinical or other personal benefits to participants.

Participation in the study is voluntary and does not involve any financial compensation or additional costs to be borne by you. You have the right to refuse to answer any question you may consider unacceptable, or to withdraw your consent to participate in the study at any time without providing a reason.

If you agree / do not agree to participate in the study and to the recording of the interview, please provide feedback accordingly.

This research has been approved by the Ethics Committee of the Faculty of Biomedical Engineering, Czech Technical University in Prague (ek@fbmi.cvut.cz). For further information, please contact the principal investigator of the study.
